# Posterior column acetabular fracture fixation using a W-shaped angular plate: A biomechanical analysis

**DOI:** 10.1371/journal.pone.0187886

**Published:** 2017-11-20

**Authors:** Ke Su, Song Liu, Tao Wu, Yingchao Yin, Ruipeng Zhang, Shilun Li, Yingze Zhang

**Affiliations:** 1 Department of Orthopaedic Surgery, Cangzhou Central Hospital, Hebei, P. R. China; 2 Department of Orthopaedic Surgery, The Third Hospital of Hebei Medical University, Shijiazhuang, Hebei, P. R. China; Mayo Clinic Minnesota, UNITED STATES

## Abstract

**Objective:**

The purpose of this study was to compare the stability and feasibility of four fixation constructs in a posterior column acetabular fracture: one reconstruction plate, one reconstruction plate and lag screw, two reconstruction plates, and a W-shaped acetabular angular plate.

**Methods:**

Twenty embalmed cadaveric pelvises with a posterior column acetabular fractures were allocated to one of four groups: 1) a reconstruction plate, 2) a reconstruction plate with a posterior column lag screw, 3) double reconstruction plates, and 4) a W-shaped acetabular angular plate. These constructs were mechanically loaded on a testing machine, and construct stiffness values were measured. Strain gauges were utilized to measure the mechanical behavior in the condition of compressive force.

**Results:**

Final stiffness was not different between the two reconstruction plates (445.81±98.30 N/mm) and the W-shaped acetabular angular plate (447.43±98.45 N/mm, p = 0.524), both of which were superior to a single reconstruction plate (248.90±61.95 N/mm) and a combined plate and lag screw (326.41±94.34 N/mm). Following the fixation of the W-shaped acetabular angular plate, the strain distribution was similar to the intact condition around the acetabulum. The parameters of the W-shaped acetabular angular plate that were observed at the superior region of the acetabulum were less than those of a single reconstruction plate (p<0.05), a single reconstruction plate with lag screw (p<0.05), and two reconstruction plates (p<0.05).

**Conclusions:**

The novel W-shaped acetabular angular plate fixation technique was able to provide the biomechanically stiffest construct for stabilization of a posterior column acetabular fracture; it also resulted in a partial restoration of joint loading parameters toward the intact state.

## Introduction

Operative reduction and internal fixation is now considered to be the treatment for unstable posterior column acetabular fractures to decrease the risk of posttraumatic arthritis and to allow early mobilization. A posterior approach (Kocher-Langenbeck) is convenient for reduction and fixation; the conventional methods of fixation often involve reconstruction plates and lag screws, or both in combination, to maintain perfect reduction. It is clear that early postoperative rehabilitation training is beneficial to joint function recovery; therefore, a rigid fixation implant that allows early mobilization is important for such fractures. In a biomechanical analysis, Schopfer et al. [1] showed that no significant differences were noted in hemipelvis posterior column osteotomies for a single 3.5-mm reconstruction plate, two such plates, and a 4.5-mm lag screw with a single plate; however, his study only loaded 550 N (0.75 times body weight) as an axial force, which did not meet the requirement of standing and walking persistently. The shortage of a biomimetic analysis for posterior column acetabular fracture is a problem that needs to be addressed. Otherwise, conventional fixation constructs mostly depend on the structure of the acetabulum and the surgical technique, as screw placement in the posterior column of acetabulum has a narrow margin of safety. All of the reconstruction plates need to be contoured into appropriate shapes according to the size of the acetabulum. Utilizing two reconstruction plates to obtain better fixation and more screws for penetration during surgery is a potentially serious traumatic complication [2,3] that may lead to the development of osteoarthritis [2].

**Fig 1:**
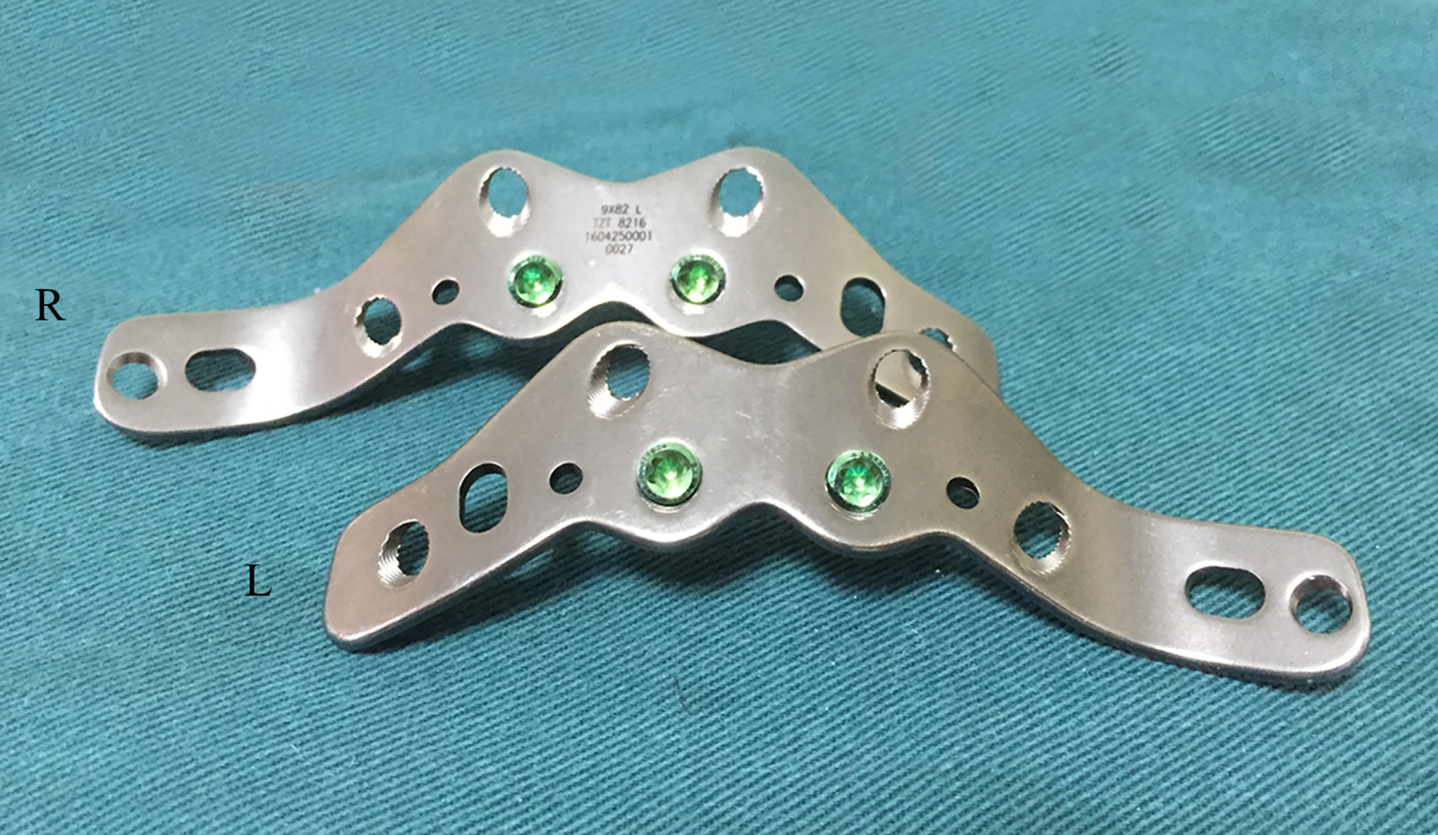
The W-shaped acetabular angular plate. R, right; L, left.

We designed a W-shaped acetabular angular plate for fractured posterior columns of the acetabulum (Fig 1; Patent No. 2009202174341). This plate-screw fixation includes a W-shaped locking plate and the guide apparatus, which have been developed to take advantage of the pre-contoured shape, extended the fix range, the oval screw hole and the angular stability. Several studies have evaluated the strength of conventional internal fixation for the posterior wall or transverse acetabular fractures [4–7]. To date, there have been no studies comparing the biomechanical stabilities of these newly developed W-shaped acetabular angular plates with conventional constructs that consist of standard pelvic reconstruction plates and lag screws, and in particular, posterior column fractures.

The purpose of this study was to evaluate the construct stability and feasibility provided by the W-shaped acetabular angular plate (WAAP) and to compare it with conventional forms of fixation that use a single reconstruction plate (1P), a reconstruction plate with a posterior column lag screw (PS) and double reconstruction plates (2P).

## Materials and methods

### Preparation and preservation of specimens

Twenty male cadaveric pelvises were provided by the Department of Anatomy of Hebei Medical University in March 2017. None of the transplant donors were from a vulnerable population and all donors or next of kin provided written informed consent that was freely given. All of the specimens were harvested from the fourth lumbar vertebra to the proximal one-third of both femurs with a layer of periosteum and the capsules of both hip joints intact. We examined all specimens visually and radiographically for evidence of abnormalities, and bone mineral density was quantified in the femoral head using a dual energy X-ray osteodensitometer (Medilink Company, Parc de la Mediterranee, France) before dissection. Only hips without signs of malignancy and arthritis were chosen for this trial. The specimens were frozen at -20°C and thawed at room temperature for 12 hours before biomechanical testing. The tissues kept moist with formalin before and during the experiments. This study was conducted in accordance with the declaration of Helsinki and was approved by the Hospital Ethics Committee of the Third Hospital of Hebei Medical University, Shijiazhuang, Hebei, China (Protocol number: National Approval No. 2015-001-1).

### Fracture of creation

**Fig 2:**
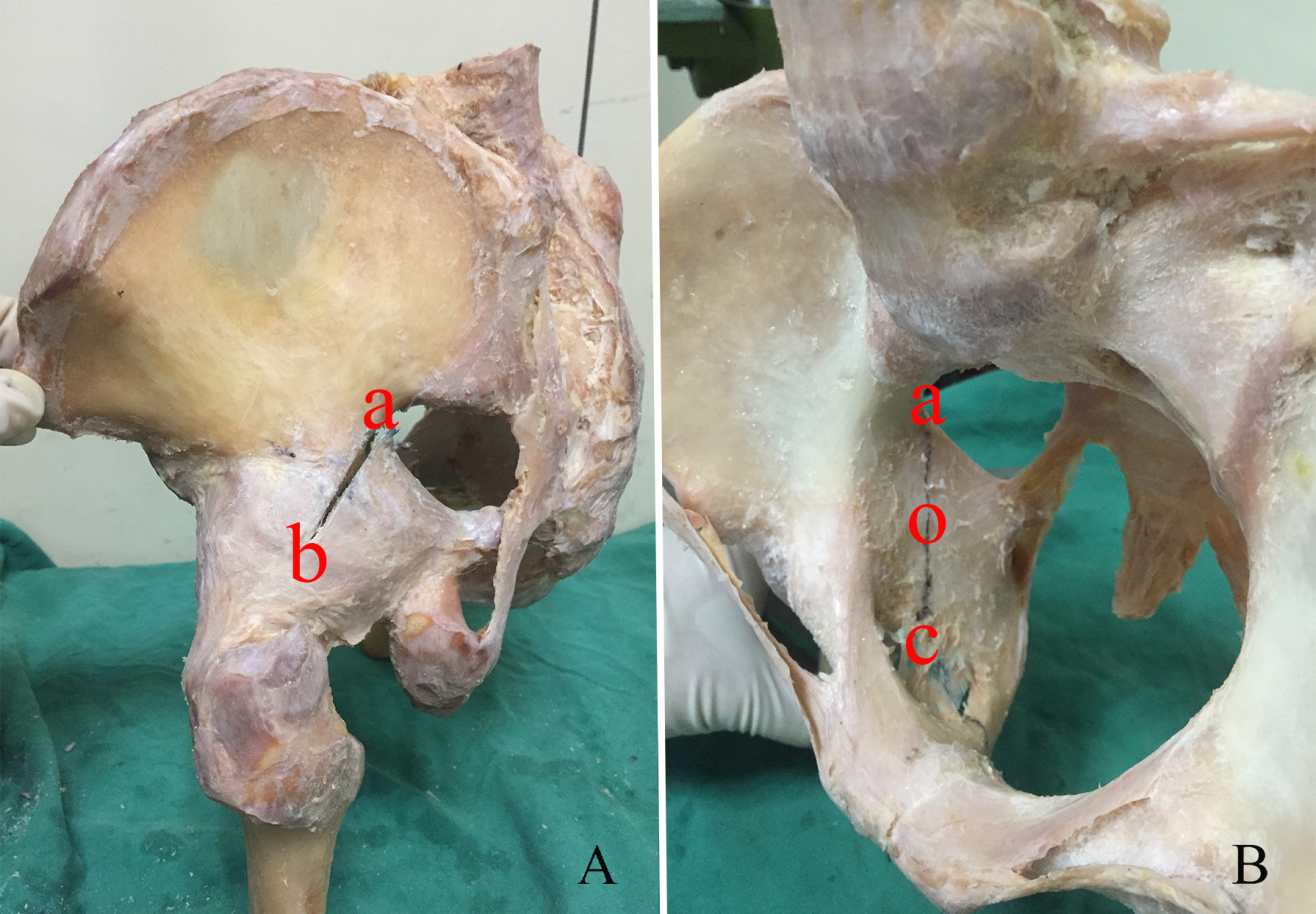
Posterior column fracture of the acetabulum. The osteotomy line originated from the vertex of the greater sciatic notch (a) to the center of the quadrilateral surface (o), the outside pelvis of the converging line went through the 2 o’clock of the left posterior acetabulum and ran along the posterior brim of incisura acetabula (c) to separate the ramus ossis ischia.

A posterior column fracture was created using an oscillating observed as previously described by Schopfer et al. [1] (Fig 2). The inside pelvis of the osteotomy line originated from the vertex of the greater sciatic notch (a) to the center of the quadrilateral surface (o), the outside pelvis of the converging line went through the posterior brim of the acetabulum, which is 2 o’clock in the left acetabulum (b) and 10 o’clock in the right acetabulum according to Knight et al. [8], and then ran along the posterior brim of the incisura acetabula (c) to separate the ramus ossis ischia. The capsules of the hip joint were kept intact.

### Structure of the WAAP

The WAAP (Tianjin Zhengtian Medical Instrument Company Ltd., Tianjin, P.R.C) is an appropriately contoured plate with a “W” shape that contains locking holes and dynamic compression holes. This plate can be placed parallel and close to the rim of the acetabulum, where it can provide the most effective buttress for the posterior column. After surgical reduction, two compression holes in the cephalic and caudal safety zone were performed first to provide the buttress roles. The directions of locking screws were dictated by the plate and were placed safely without C-arm visualization. All screws had been confirmed to be in an extra-articular location and of an appropriate length.

### Instrumentation

**Fig 3:**
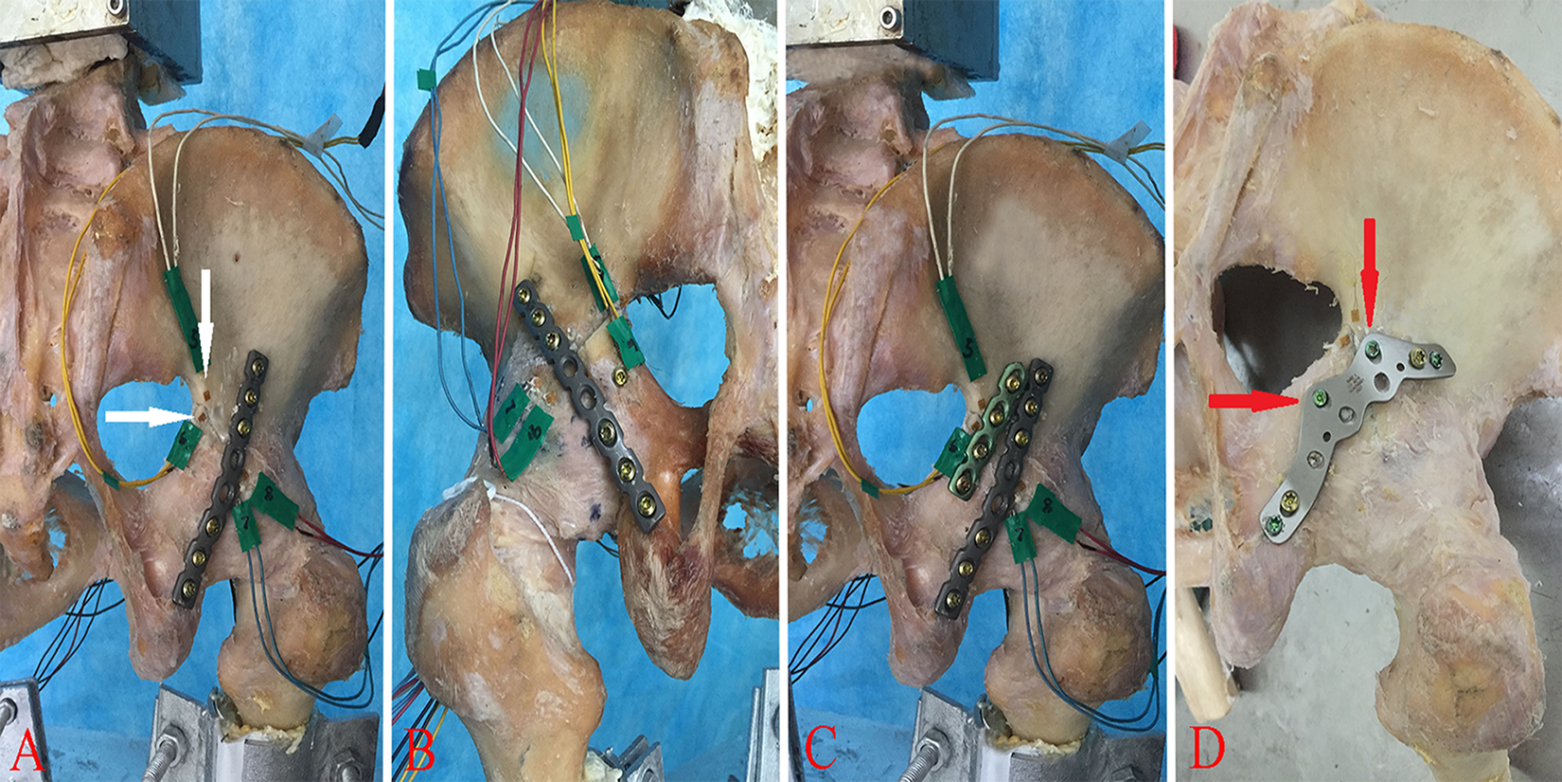
Groups of the different fixation constructs tested. (A) One 8-hole 3.5-mm reconstruction plate with six bicortical screws. (B) One 8-hole 3.5-mm reconstruction plate with six bicortical screws supplemented with one 4.5-mm posterior column lag screw. (C) One 8-hole 3.5-mm reconstruction plate with six bicortical screws and one 4-hole 3.5-mm reconstruction plate with four bicortical screws. (D) One 7-hole WAAP with two bicortical screws and four locking screws. White arrow, strain gauges; Red arrow, two locking screws in the medial column of the WAAP pointed towards the greater sciatic notch.

All specimens were randomly divided into four groups, and the osteotomies were reduced anatomically in the four groups and fixed in one of four ways (Fig 3): (1) one 8-hole posterior column 3.5-mm reconstruction plate with three bicortical screws on either side of the osteotomy (group 1P); (2) one 8-hole posterior column 3.5-mm reconstruction plate with three bicortical screws on either side of the osteotomy supplemented with one 4.5-mm posterior column lag screw (group PS); (3) one 8-hole 3.5-mm reconstruction plate with three bicortical screws plus one 4-hole 3.5-mm reconstruction plate with two bicortical screws on either side of the osteotomy (group 2P); and (4) one 7-hole WAAP with one bicortical screw and two locking screws on either side of the osteotomy (group WAAP). The lengths of the nonlocking cortical screws ranged from 20–55 mm in the reconstruction plates, and the lengths of the locking screws ranged from 20–45 mm in the WAAP. Fluoroscopy was used to confirm appropriate hardware application and fracture fixation.

### Biomechanical testing

#### Displacement measurement

**Fig 4:**
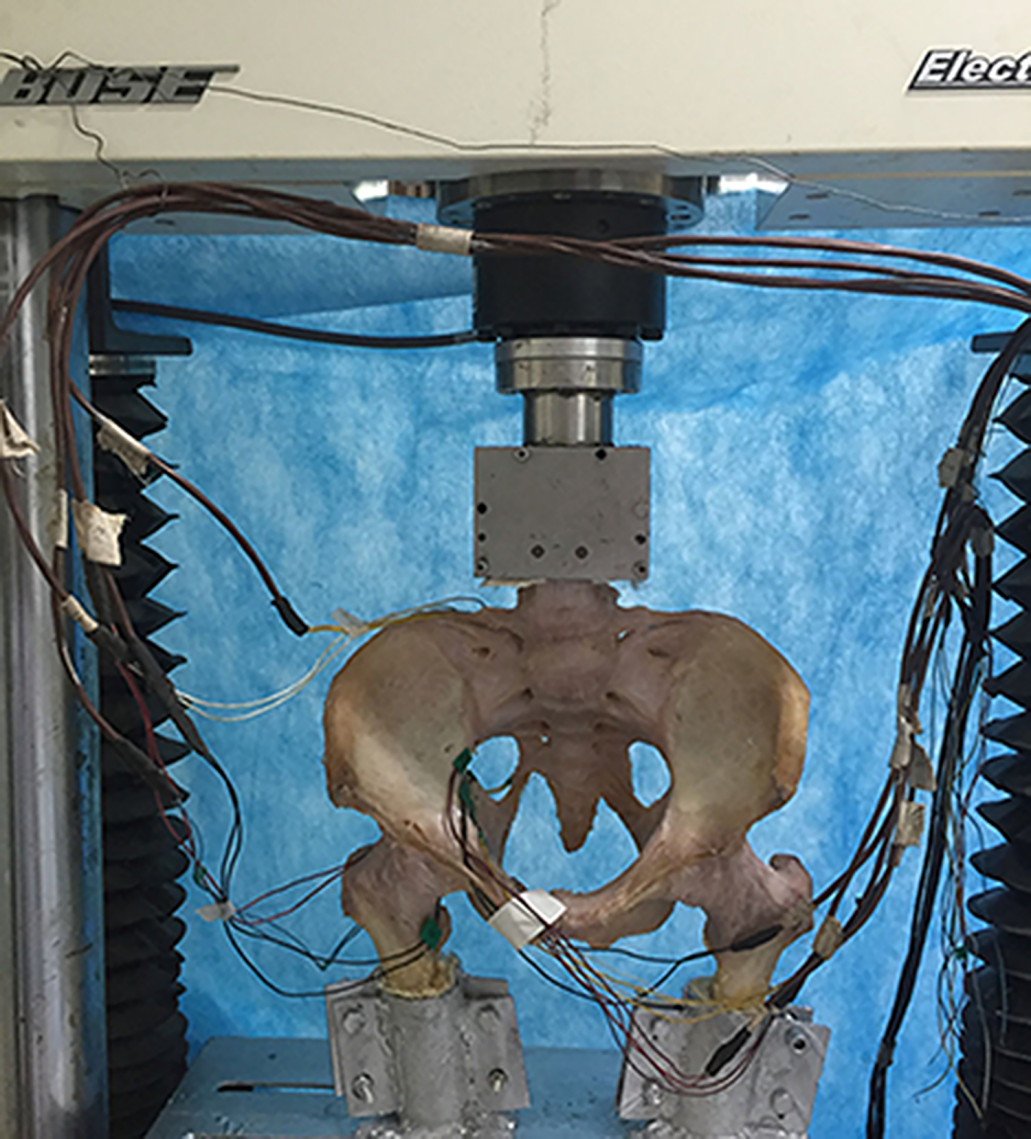
The load cell and jig used to position the pelvis and femur. The pelvis is mounted in a double-limb standing position, and axial load is applied through the fourth lumbar vertebra.

We adopted a testing methodology similar to the one previously published by Sawaguchi et al. [9], in which the specimen was mounted in a double-limb standing position and was supported distally by bilateral femur (Fig 4). The pelvic specimens were loaded in an Electroforce 3520-AT Bose biomechanical testing machine (BOSE Corporation, Eden Prairie, USA). The anterior superior iliac crest was parallel to the pubic tubercles, the femoral head was directed at 45° of abduction and 15° of internal rotation, the distal femurs were potted in separate epoxy resin blocks (Shanghai, Medical Instrument Company Ltd, Shanghai, P.R.C.), and the loading apparatus was via the fourth lumbar vertebra. Theoretically, we estimated approximately 2 times the body weights for double-limb standing position (1400 N). After internal fixation was implanted, a ramp waveform was applied at the rate of 10 N/s up to 200 N to eliminate creep. Then, the cyclic loading protocol was between 0 and 1400 N at 1 Hz with 10 cycles. The stiffness and vertical compression displacement were quantified using Wintest7 Software. All 20 constructs survived, and no evidence of permanent deformation or failure was noted.

#### Dynamic strain measurement

**Fig 5:**
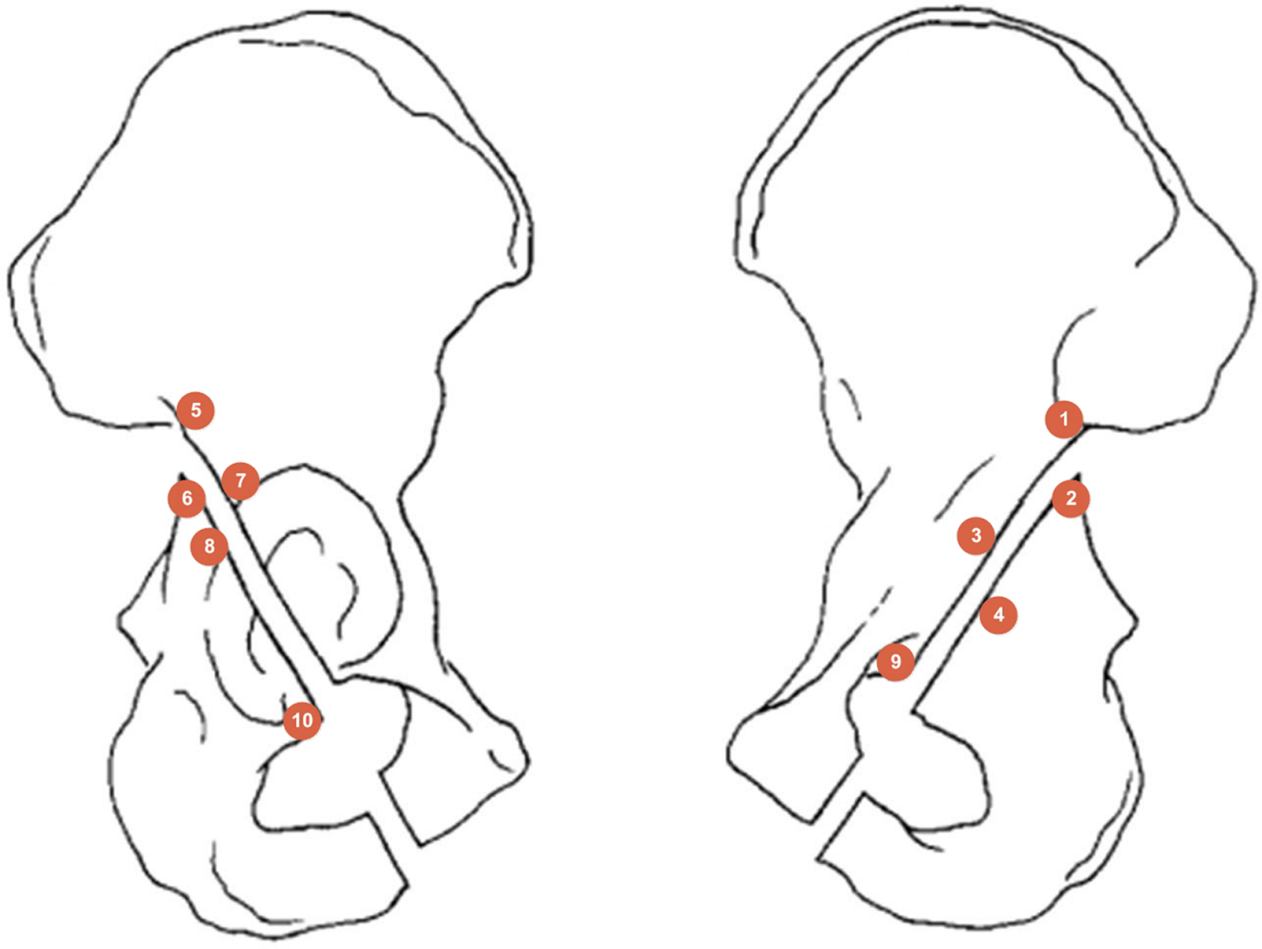
Positions of the strain gauges at 1–10 points around the acetabulum.

Strain gauge was a simple procedure to evaluate the stress distribution in the cortical bone [10]. We defined 10 points around the acetabulum as depicted in Fig 5. Initially, we measured the strain distribution of 20 intact pelvises and recorded the parameters in the superior (point 2), posterior (point 8), inferior (point 10) and interior (point 4) regions. Then, the posterior column fracture was created, and the model was instrumented with ten pieces of tri-axial strain gauges (BHF350-3AA, Yunhui technology Co., Ltd, Shenzhen, P.R.C.) placed at a 45° angle on both sides of the osteotomy line. The surface sites were previously prepared to remove the periosteum approximately 0.5 cm^2^ from all contact regions by sanding, grinding, wiping with ethanol and pasting strain gauges with glue (Fig 3A). They were then linked to a measuring instrument (Wavespectrum, Beijing Wavespectrum Science and Technology Co., Ltd, Beijing, P.R.C.) with signal acquisition software (Vib’SYS Software) and recorded at the load of 1400 N. Each pelvis was loaded for 10 successive trials, and the strain distributions were measured at the gauges. This dynamic strain measurement was performed simultaneously with displacement measurement.

### Statistical analysis

The sample size estimation was calculated with Power and Sample Size Calculators (http://powerandsamplesize.com/; HyLown Consulting LLC, Atlanta, GA) using the biomechanical data published by Mehin et al. [11]. Their stiffness means and variance estimates were taken from fresh-frozen pelvises with simulated acetabular fractures instrumented with conventional or locking plate fixation. A total sample size of 18 was required for this study assuming a false-positive rate of 5% (*α* = 0.05) and a power of at least 80% (*β* = 0.20); therefore, the experimental design required a valid sample size of 5 per group.

We analyzed differences in specimen stiffness, strain distribution of intact state and tolerable strains with the four methods of fixation when loaded to 1400 N. Stiffness was defined as the slope of the force versus the displacement curve. Strain gauges were deformed, the resistance of wire gauze changed along with different compressive forces, and the electrical signal parameter was converted into a digital quantity. The data were reported as the means and standard deviations or frequencies. Normality was confirmed using the Shapiro-Wilk test. The quantitative data were compared by one-way analysis of variance (ANOVA) followed by the Bonferroni test when the variances were homogenous or the Welch and Tamhane’s T2 test when the variances were not homogenous. All analyses were conducted with SPSS 17.0 at a significance p value of 0.05.

## Results

### Analysis of construct stiffness

**Fig 6:**
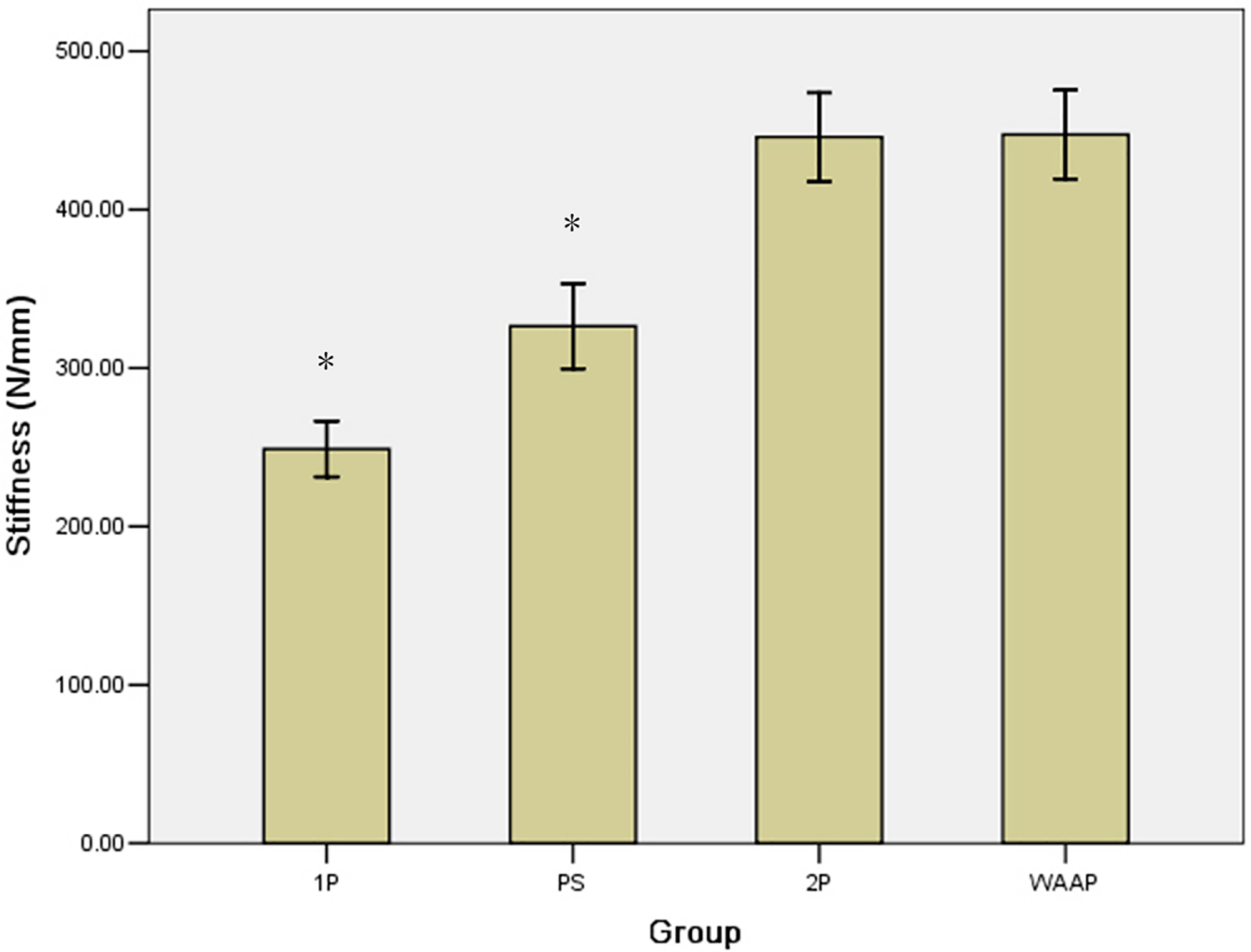
The mean stiffness values for the four different constructs. The 2P and WAAP constructs were stiffer than the 1P and PS constructs. * Different from WAAP (p<0.05).

The stiffness values of all of the repaired posterior column acetabular fracture constructs were measured by the motion of the pistons of the material testing machine when 1400 N of compression force was applied. The results presented a continuous linear behavior between different load levels in all pelvises (Table 1). A construct with two reconstruction plates (2P) (445.81±98.30 N/mm) was stiffer than one with a single reconstruction plate (1P) (248.90±61.95 N/mm, p<0.05) and was stiffer than the single reconstruction plate with one lag screw (PS) (326.41±94.34 N/mm, p<0.05). The WAAP (447.43±98.45 N/mm) was also stiffer than the single reconstruction plate (1P) (p<0.05) and stiffer than the single reconstruction plate with one lag screw (PS) (p<0.05). The constructs in the 2P and WAAP groups displayed the highest stiffness values, and there was no significant difference between them (p = 0.524). The construct with the single reconstruction plate with one lag screw was stiffer than the single reconstruction plate alone (p<0.05) (Fig 6).

**Table 1:** Mean stiffness values and standard deviations for different fixation modalities at the load of 1400 N.

Group	Stiffness (N/mm)^a^	
	Mean±SD	95% CI
1P	248.90±61.95	231.30–266.51^b^
PS	326.41±94.34	299.60–353.22^c^
2P	445.81±98.30	417.87–473.75^d^
WAAP	447.43±98.45	419.45–475.41

^a^ Stiffness analysis of variance F = 58.73, p = 0.000

^b^ p<0.05 compared with 2P and WAAP

^c^ P<0.05 compared with 2P and WAAP

^d^ p = 0.524 compared with WAAP

CI, confidence interval; 1P, one reconstruction plate; PS, one reconstruction plate with one lag screw; 2P, double reconstruction plates; WAAP, W-shaped acetabular angular plate.

### Analysis of construct strains

**Fig 7:**
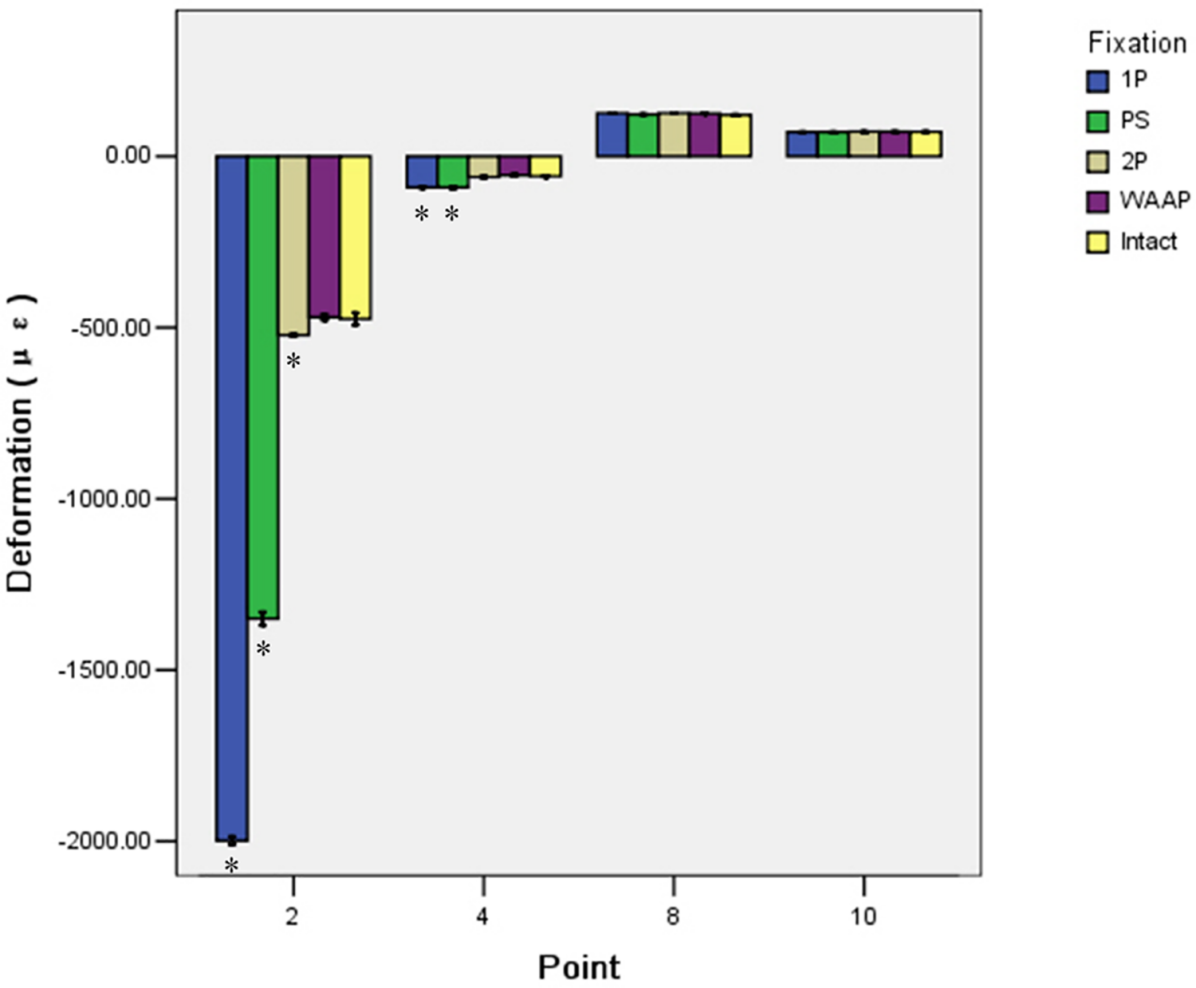
Mean deformations for the four different constructs compared with the intact state. There were significant differences from the intact condition in both the superior (point 2) and interior (point 4) regions. * Different from intact (p<0.05).

The experimental strains were on averaged from ten cycle measurements made for each load. Table 2 presents the strains and standard deviations for each tri-axial strain gauge of the pelvis. The ten points were measured from four regions: superior (points 1, 2, 5, and 6), posterior (points 7 and 8), inferior (points 9 and 10), and interior (points 3 and 4). After 1400 N was applied, all constructs demonstrated that the principal strain distribution was presented in the superior of the acetabulum, especially around the vertex of the greater sciatic notch. In the superior regions, points 1, 5, and 6 showed a significant difference among the four fixation schemes (p<0.05). We compared the four fixations to the intact condition in point 2. The WAAP construct displaced the minimum strain deformation and was no different from the intact state (p = 1.000). In the posterior and interior regions, there was no significant difference between the 2P and WAAP groups, but they were superior to the 1P and PS groups. In the inferior region, the strain distributions were all close to that of the intact state and were not different between the four constructs (p = 0.086) (Fig 7).

**Table 2:** Mean strains and standard deviations for different fixation modalities at the load of 1400 N.

Point	Regions	Deformation (με)				
		1P	PS	2P	WAAP	Intact
1	Superior	2190.34±198.73	1570.56±139.47	476.66±36.19	468.44±36.96^a^	
2		-1998.44±37.40	-1350.02±65.92	-522.22±15.66	-469.84±27.62^a^	-474.49±120.29
5		2154.64±91.81	1508.90±68.91	528.02±15.07	422.80±21.38^a^	
6		-1896.72±40.30	-1245.94±56.84	-533.02±7.26	-433.84±11.51^a^	
7	Posterior	-121.26±3.05	-121.94±4.05	-121.30±2.76	-120.12±2.88^a^	
8		126.02±3.14	122.48±4.27	126.06±5.86	124.88±5.88	120.92±11.30
9	Inferior	41.34±1.87	41.28±2.25	42.28±2.43	41.86±2.58	
10		71.42±1.90	71.28±2.25	72.34±2.19	72.02±1.85	71.60±10.41
3	Interior	125.94±12.09	125.32±14.36	89.66±8.99	91.74±11.27	
4		-90.72±5.47	-90.94±10.10	-60.22±7.42	-55.26±10.53^a^	-58.06±16.73

^a^ Deformation was significantly decreased in the WAAP group compared with the other groups (p<0.05), there was no difference from the intact state at point 2 (p = 1.00).

1P, one reconstruction plate; PS, one reconstruction plate with one lag screw; 2P, double reconstruction plates; WAAP, W-shaped acetabular angular plate.

## Discussion

The important finding of this cadaver study was that the WAAP fixation was stiffer than a single conversion plate and lag screw. In particular, the WAAP fixation displaced the minimum deformation at the superior of the acetabular osteotomy line. It is essential to choose the appropriate fixation method in clinical practice. As is well known, acetabular fractures remain one of the most difficult orthopedic injuries to treat surgically, as even the slightest defect (>1 mm) in the articular surface may lead to posttraumatic arthritis and poor functional outcomes [12,13]. Conventional fixations of posterior column acetabular fractures involved one or two reconstruction plates and/or one reconstruction plate with one lag screw [14,15]. However, the reconstruction plate and lag screw may not be rigid enough, and the surgical technique and the surgeon’s experience level should be taken into consideration when confirming that no screws are penetrating the hip joint [16]. We designed the WAAP for posterior column acetabular fractures to avoid these problems. The shape of the plate and the safe angle of the locking hole were based on our previous study [17]. Four types of the WAAP were designed according to the body heights (1.51 to 1.60 m, 1.61 to 1.70 m, 1.71 to 1.80 m and 1.81 to 1.90 m), each had its own shapes and safe angles and was best suited to different size of acetabulum. In the actual application, we chose the well-sized WAAP in congruence to the contour of the danger zone, with the outer edge of WAAP parallel to the lateral acetabular brim, which could make sure the screws would not penetrate into the hip joint. As new fixations are introduced, studies are needed to compare their biomechanical strength with constructs that are well established. Our studies aimed to determine if this WAAP fixation conferred similar stability to traditional forms of fixation.

For this study, we found that the overall amount of displacement among the four groups was relatively small and that main direction of the fragment movement was in the shear direction. Therefore, we decided to consider the overall structure displacement due to the motion of the pistons of the material testing machine divided by the compressive force as the construct stiffness, with the actual load plane lying 60º anterior to the fracture plane” as an alternative here [1]. Otherwise, the position of the double-limb stance and the capsules of both intact hip joints had both advantages and disadvantages;–this position could simulate a neutral standing posture as close as possible and avoid the rotation of the iliac crest [18]. However, nearly half of the compressive force would load on the normal side of the acetabulum. Otherwise, displacement of the sacroiliac joint and hip cartilage deformation would be involved in the recorded displacement. Therefore, first we compared the deformations in all of the intact specimens without osteotomy lines, and there was no significant difference in the four intact groups.

Our results revealed that 2P fixation constructs and WAAP constructs were significantly stiffer than those in the 1P and PS groups. There was no statistical stiffness difference between the 2P and WAAP groups. The addition of a 4-hole 3.5-mm reconstruction plate would be equal to the two pieces of locking screws in the medial column of the WAAP (Fig 2D), which were located on both sides of the osteotomy line and pointed to the greater sciatic notch. Several biomechanical studies have indicated that locking screws can provide more stability than a conventional construct [19,20], which is the same as our results. Although the conventional two reconstruction plate construct provided the same stiffness as the WAAP, its disadvantages included insufficient contouring to adapt plate-bone contact and friction to achieve stability. Perfect contouring is much easier in cadaveric samples than in vivo. Furthermore, the two-plate construct may cause screw interference.

In our study, strain gauges were used to measure the principal strain distribution. There was a proportionality between load and principal strains. Although this proportionality was not constant on the entire surface, the average of the principal strains can be considered as proportional to the load case. The strains were more pronounced on the superior regions, consistent with the study published by Dastra et al. [21]. After the posterior column acetabular fracture was created, the interruption of the posterior column continuity led to blockage of the normal pressure transmission. The conventional reconstruction plate and lag screw cannot result in a restoration of joint loading parameters toward the intact state because of the insufficient width. However, the 2P and WAAP constructs increased the posterior column cover area and provided an effective buttress to reduce cortical bone deformation.

In a clinical situation, the WAAP provides some important advantages compared with two reconstruction plates, including a more effective means of buttress plating the posterior wall and spanning the fixation to the whole posterior column [22]. First, the extended fixation range spans from the greater sciatic notch to the rim of the posterior acetabulum. There are two rows of drill holes in the danger zone region. Depending on the fracture pattern, we have a different procedure that can be used to ensure stability. Second, the WAAP is anatomically pre-contoured and could match the surface of the posterior acetabulum column properly so that minimal intra-operative bending would be required. Third, the WAAP has locking holes to achieve angular stability. Avoidance of screw placement in the danger zone of the acetabulum could minimize the possibility of screw penetration into the hip joint. Actually, this uniquely angled design of the safe-angled drilling guide removes the necessity of evaluating complications, regarding not only fixation in the posterior of the acetabulum but also maintaining the overall stiffness and facilitating the operation.

This study had several limitations. First, we chose to use embalmed cadaveric pelvises with a sample size of five per group. Although bone mineral density was quantified before biomechanical testing, the small sample size and the specimen quality levels were different, which could influence the accuracy of the experiment. Second, the theoretical fragment movement is in the three directions: horizontal, lateral and vertical. Therefore, the displacement measured by the motion of the piston did not accurately represent the movement–just a part of the actual movement. Therefore, we accurately located the position, and a ramp waveform was applied before the start of the vertical compression loading to eliminate creep. Third, we kept the anterior column of the acetabulum intact and created a posterior column fracture in theory. Actually, the posterior column acetabular fracture was usually accompanied by the pelvic ring fracture, such as the T-Shaped acetabular fracture and two column acetabular fracture. It is crucial to achieve the stable fixation for the two column of acetabulum, which may have an effect on the entire biomechanics of the acetabular fracture.

## Conclusions

The novel WAAP fixation was able to provide the biomechanically stiffest construct and resulted in a restoration of strain distribution for posterior column acetabular fracture. Furthermore, this fixation system entails a wide range of fixation, a pre-contoured shape, and angular stability. Further studies are required to assess the value for more clinical posterior column acetabular fracture fixations.

## Supporting information

S1 Table Mean stiffness values and standard deviations for different fixation modalities at the load of 1400 N. ^a^ Stiffness analysis of variance F = 58.73, p = 0.000; ^b^ p<0.05 compared with 2P and WAAP; ^c^ P<0.05 compared with 2P and WAAP; ^d^ p = 0.524 compared with WAAP; CI, confidence interval; 1P, one reconstruction plate; PS, one reconstruction plate with one lag screw; 2P, double reconstruction plates; WAAP, W-shaped acetabular angular plate.
(XLSX)

S2 Table Mean strains and standard deviations for different fixation modalities at the load of 1400 N. ^a^ Deformation was significantly decreased in the WAAP group compared with the other groups (p<0.05), there was no difference from the intact state at point 2 (p = 1.00). 1P, one reconstruction plate; PS, one reconstruction plate with one lag screw; 2P, double reconstruction plates; WAAP, W-shaped acetabular angular plate.
(XLSX)
