# Correction: Posterior column acetabular fracture fixation using a W-shaped angular plate: A biomechanical analysis

**DOI:** 10.1371/journal.pone.0217734

**Published:** 2019-06-05

**Authors:** Ke Su, Song Liu, Tao Wu, Yingchao Yin, Ruipeng Zhang, Shilun Li, Yingze Zhang

There is an error in affiliation 1 for author Ke Su. The correct affiliation 1 is: Department of Microbiology, Tumor and Cell Biology (MTC), Karolinska Institutet, Stockholm, Sweden.
