# Correction: Correction: Posterior column acetabular fracture fixation using a W-shaped angular plate: A biomechanical analysis

**DOI:** 10.1371/journal.pone.0218646

**Published:** 2019-06-13

**Authors:** The PLOS ONE Staff

There is an error in affiliation 1 for author Ke Su in the correction published on June 5, 2019. The correct affiliation 1 is: Key laboratory of biomechanics of Hebei Province, Hebei Medical University, Shijiazhuang, Hebei, P. R. China. The publisher apologizes for the error.
